# The effects of cultural dimensions on export performance: Vietnam and Colombia cases

**DOI:** 10.1016/j.heliyon.2022.e11785

**Published:** 2022-11-25

**Authors:** Diana Escandon-Barbosa, Jairo Salas-Paramo

**Affiliations:** Gestion de Organizaciones Department, Pontificia Universidad Javeriana, Cali, Colombia

**Keywords:** Ambidextrous leadership, Export performance, Organisational paradox, Exploitation, Exploration, Born global, *JEL:*, M13, L26

## Abstract

This research focuses on the moderation effects of Hofstede's cultural dimension that influence the relation between ambidextrous leadership and export performance in Born Global Firms in two countries with different cultural characteristics, Vietnam and Colombia. We collected the data from 800 exporting firms in two countries, Colombia and Vietnam, that are characterised by their having begun international operations in the first three years of being Born Global. The results demonstrate that ambidextrous leadership has a positive impact on export performance. Additionally, the dimensions of uncertainty avoidance and indulgence moderate the relation between ambidextrous leadership and export performance, while long-term orientation does not affect the relationship.

## 1 Introduction

Latin America and the Caribbean SMEs are around 95% of the companies and employ approximately 67% of those working. They contribute less than a third of the GDP. Additionally, their export dynamics have been characterised by selling few products to few markets (González-Uribe and Reyes, 2019). In contrast, to European companies, Born Global (BG) represents 40% of small and medium-sized companies in all of Europe and 12% of young companies. They have contributed significantly to employability and the ability to mitigate economic recessions (OECD, 2013; Eurofound, 2012; Eurofound, 2018; Lafuente et al., 2018).

Born Global firms, also known as Born Global companies (BGs), that develop activities from the early stages of their life cycle, are significant because of their international nature. They combine capacities and resources both in creating a business and in the inclusion in the international market (Escandon-Barbosa et al., 2019; Weerawardena et al., 2019; Prieto -Sánchez & Merino, 2021).

In the field of BGs, it is possible to find three critical gaps in the objectives BG is trying to reach. The first is the need to analyse the environmental factors that influence the performance of a BG, such as its cultural aspects. The second gap concerns the importance of using resources when resources are scarce. The third and final gap is the need to conduct studies between countries with cultural differences and different environmental conditions. The first gap is related to the environmental aspects that most influence a BG's dynamics (da Rocha et al., 2017).

The first gap is also related to the influence of the environment on a BG's performance. The discrepancy in cultural, economic, and environmental elements that influence BGs, especially in rising economies like Latin America, is proof of this (Baier-Fuentes et al., 2019; Paul and Rosado- Serrano, 2019; Perényi and Losoncz, 2018). Considering these facts, we attempt to fill the gap in how cultural aspects influence the emergence and dynamics of BGs in Latin America.

The second gap in the field of BG is the need to analyse how BGs can use their resources to carry out innovation processes. The reasons for this gap carry a set of tensions that directly affect how BGs experience pressure. Finally, the third gap is related to the need to conduct comparison studies between countries to identify their main differences (Ramirez and Tadesse, 2009).

In this approach, the current study aims to fill in some of the gaps that have been identified, particularly in investigating environmental factors that influence Born Global Firms. This analysis considers the moderating effects of Hofstede's cultural dimensions (uncertainty avoidance, long-term orientation, and indulgence/restraint), which have been selected as the dimensions in which the two countries have different levels of development according to Hofstede's Insight.

Similarly, an attempt is made to analyse the influence of ambidextrous leadership on export performance to identify possible differences in the leadership styles assumed in the firms. This influence is how the firms' resources and capacities are used to generate an impact on the results of the born global firms. Finally, an attempt is made to contribute to the realisation of comparative studies between countries with different cultural characteristics.

To achieve the objectives of the research, we have structured this paper as follows: in the first part, we contextualise BGs' dynamics related to export performance and the primary research needs in the field. This condition is followed by an analysis of literature on ambidextrous leadership and Hofstede's cultural dimension highlighting the main results in the field. In the third part, we present the methodology and the main results. Finally, we discuss the study's conclusions.

## 2 Literature review

### 2.1 Export performance

Much of the literature on born global firms have focused on the relationship between different strategies and their effects on export performance (Di Fatta, Gera, Tyagi and Grisold, 2019; Aaby and Slater, 1989; Cavusgil and Zou, 1994). This research considers the definition of performance as a process related to how companies use their capabilities to be more effective than their competitors. Additionally, this definition considers the satisfaction of the needs of their clients to improve their competitiveness in an international context (Ngo-Thi-Ngoc & Nguyen-Viet, 2021; Monteiro et al., 2019).

Aaby and Slater (1989); propose a model in which internal and external factors mainly constitute the export performance. The external factors are those located at the macro-economic, social, physical, cultural and political levels that directly influence export performance (Di Fatta, Gera, Tyagi and Grisold, 2019).

These external factors are related to the conditions in which the manager's actions are executed. On the other hand, the internal factors are three firm competence, firm characteristics and export strategy. Later in the literature, finding other variables such as technology, market knowledge, planning export policy, management control, and communication has also been possible. On the other hand, the literature raises some characteristics of the firms, which are associated with size, managers' commitment, and attitudes to export (Di Fatta et al., 2019).

Other scholars, such as Cavusgil and Zou (1994), argue that export performance is higher when commercial strategies are deliberate in how they are implemented. In the same way, they show how external and internal factors that influence export performance depend indirectly on the marketing strategy. Regarding marketing strategies, field studies have found this factor conditions of success that allow companies to export under more favourable conditions.

According to Di Fatta et al. (2019), studies in the field of export performance suggest that there are no single factors that influence it, but instead that there are different approaches that allow success in this type of international operation. One of the main weaknesses is that there is no consensus or unification of the conclusions due to the short synthesis and assimilation of fragmented knowledge (Katsikeas and Leonidou, 1996). In this condition, export performance should be explored from the antecedents that can be operationalised and measured differently.

These measurements of the concept consider objective and subjective approaches so that it allows assuming variables to explain the different variations in export performance. Among these variables are export sales which refer to the size of profits in financial terms (Madsen, 1989). On the other hand, export intensity is considered the ratio of total sales and sales of the firm (Axinn and Thach, 1990; Zou et al., 1998). Export growth refers to export made during a specific range of time. Moreover, export profitability refers to the percentage of the firm's total profits in export activities.

It is important to note that the literature in the field of export performance indicates the importance of carrying out studies on the influence of behaviours on export performance. This type of behaviour can be assumed as an antecedent of the export performance that affects the export results of the firms (Katsikeas and Leonidou, 1996).

### 2.2 Ambidextrous leadership perspective

According to Jia et al. (2021), in managerial practice, there are a series of tensions between two types of leadership situations whose main objective is the integration and coordination of two contradictory and complementary leadership styles. This leadership style is called Closing leader behaviour and opening leader behaviour. In the closing leader behaviour, the leader focuses on sanctions for mistakes that may be made in dynamic organisations. At the same time, the opening leader behaviour pays attention to the tolerance processes in the different actions that may take place within the firm (Miron-Spektor et al., 2018; Lewis et al., 2014).

The literature on ambidextrous leadership establishes that the concept includes the coordination between a set of various behaviours and leadership styles (Tuan, 2017; Zacher and Rosing, 2015). Therefore, ambidextrous leadership requires consistency between opposing behaviours (Rosing et al., 2011; Luo et al., 2018).

Ambidextrous leadership is directly related to the context in which the firm develops its activities (Luu, Dinh & Qian, 2019). From a theoretical point of view, ambidextrous leadership considers situational contingencies which allow an effective response to different phenomena presented in the market (Rosing et al., 2010). This type of response allows identifying a relationship between contingency leadership theory and ambidextrous leadership. This kind of response shares the idea of facing the environment's dynamics through behaviour with flexibility and adaptability characteristics (Luu et al., 2019).

Ambidextrous leadership focuses on how the behaviours of individuals develop the ability to respond to changes in goals and tasks established over time (Rosing et al., 2011). The fundamental premise of ambidextrous leadership is based on the approach in which the actions that corporate leaders carry out to stabilise situations and restore stability to the organisation (Luo et al., 2018).

Regarding export performance, the organisation is permanently exposed to constant uncertainty that requires open and progressive behaviours to adapt to market demands (Luo et al., 2018). Competitive environments characterise markets that born global firms serve. This condition limits the possibility of having enough time to think about what type of leadership is the best and adopt it. Therefore, leaders who show ambidextrous become facilitators of change in strategies focusing on exploitation as exploration (Jansen et al., 2009).

Export performance is directly related to exploration, characterised by a focus on experimentation, assuming risks and searching for possible solutions to market situations or phenomena. In this way, this type of leadership leads people to think openly, be more autonomous and strive to overcome the challenges imposed by new markets (Rosing et al., 2011). According to Duncan (1976), innovation can be generated from various structural agreements arising from the interaction between exploitation and exploration if they are under ambidextrous leadership. This is how much research has shown a direct relationship between ambidextrous leadership and firm performance (Zacher and Rosing, 2015). Therefore, we propose the following hypothesis:

*H1* *Ambidextrous leadership has a positive impact on export performance in Vietnam.*

*H1a* *Ambidextrous leadership has a positive impact on export performance in Colombia.*

### 2.3 Hofstede's cultural dimensions

One of the most influential authors in the analysis of cultural factors and their impact on organisations at the country level has been Hofstede. For him, a set of dimensions related to cultural aspects explain companies' performance (Hofstede, 2001; Hofstede and Hofstede et al., 2005; Hofstede et al., 2010). With these dimensions, cultural aspects can generate the conditions for success in business operations at an international level. In this case, said variables would explain the cultural differences between countries that impact the internal dynamics and their operation's external conditions (Escandon-Barbosa et al., 2021; Gallego-Álvarez & Pucheta-Martínez, 2020).

One of the most critical aspects of Hofstede's work is the origin of the model that he started with IBM employees and its subsequent inference at the level of national cultures (Hofstede, 1997). This model initially consisted of five dimensions: power, uncertainty avoidance, identity, gender, and time orientation. Recently, he added a sixth dimension, called an indulgence.

One of the Hofstede dimensions we are considering in the present study is long-term (time) orientation. This condition alludes to an aptitude oriented towards a future reward. This dimension has its opposite side called short-term orientation, whose aptitude is focused on the past and the present, having as its central characteristic respect for tradition and social obligations (Hofstede, 2001). Hofstede pointed out that the long-term orientation allows companies to assume an orientation to achieve a strong position in the market. In this dimension, the company does not expect immediate results; on the contrary, it allows them to observe the inclination of a society towards a reward over time (Guo et al., 2018).

### 2.4 Hofstede's dimensions (Vietnam and Colombia)

The specific cases Colombia and Vietnam are two countries that have different cultural characteristics. According to Hofstede Insight, countries like Colombia have dimensions with different dynamics. In the case of the two countries, the dimensions that present valuations that present the most significant differences are a long-term, indulgence, and uncertainty avoidance (Hofstede Insight, 2021).

Additionally, Vietnam has improved in the transformation into a market economy since the ‘90s, characterised by progressive industrialisation and modernisation (Cakanlar and Nguyen, 2019; Cho et al., 2014). Some studies have carried out studies with dimensions such as uncertainty, power distance, and masculinity compared with countries such as Sweden and Turkey in shopping behaviour studies (Sharma et al., 2010). However, the variables considered in the present study, especially comparative studies between countries with different characteristics, have been scarce (Cakanlar and Nguyen, 2019).

In the case of Colombia, studies have shown that the evasion of uncertainty is high. Much of the rules established in compliance are not directly related to the existence contrary to countries with Asian cultures. Additionally, Colombia is characterised as a country with a tendency toward masculinity in the short term (Guinand and Arenas, 2020).

Ambidextrous leadership is an essential factor for a company to achieve long-term success. However, its relationship with the long-term orientation has attracted noticeable attention since it is considered a critical capacity for a firm, but it is difficult to achieve (Wei et al., 2014; March, 1991; Levinthal & March, 1993; Tushman and O'Reilly, 1996; He and Wong, 2004; Laureiro-Martínez et al., 2014). Some scholars, such as Wei et al. (2014), have stated that one of the most significant difficulties in achieving ambidextrous leadership is using resources to improve the firm's performance. On the other hand, other authors have proposed that exploitation and exploration are incompatible due to the scarcity of resources (March, 1991, Uotila et al., 2009). For authors such as Ocasio (1997, 2011), a firm's ability to use the conditions of its resource will depend on the value system in which the company is immersed. Another essential element to highlight is that Wei et al. (2014) have called for studies to include cultural variables and their effect on ambidextrous leadership and the firm's performance. Finally, the literature proposed that countries characterised by long-term preference will privilege perseverance and future rewards.

In contrast, countries with a short-term orientation will give greater importance to past and present events about social traditions and obligations. According to Hofstede (2001), Asian countries tend to be oriented toward the long-term, while Western countries, such as the United States and Latin America, identify themselves as short-term oriented. Therefore, countries with more long-term orientation will have greater success in ambidextrous leadership and its contribution to the company's export performance. Therefore, we propose the following hypothesis:

*H2* *Countries with a high long-term orientation will positively affect the relationship between ambidextrous leadership and export performance.*

*H2a* *Countries with a low long-term orientation will have a lower positive moderation effect on the relation between ambidextrous leadership and export performance.*

Hofstede defined indulgence as a gratification of basic human desires related to enjoying life (Escandon-Barbosa et al., 2021). When societies are characterised as indulgent, they tend to be more permissive about the pleasures and fun in life. This society tends to prioritise freedom, leisure, and happiness. Therefore, tolerant societies will prefer happiness and tend to have broader perceptions of freedom, health, and control over life (Ismail and Lu, 2014). Despite this, there are few studies on indulgence and its effects on countries with these characteristics.

According to Guo et al. (2018), indulgence is given from strict social norms that influence people's behaviours. They added that indulgence is greater in American countries than in Asian countries, thus generating orientations towards restrictive behaviours. According to Kedmenec and Strašek (2017), people from an indulgent culture tend to be characterised by functional activities more so than less indulgent countries. Therefore, considering that the social integration between companies gives the ability to adapt to market dynamics and effectively meets the needs of the environment, a country with greater indulgence will have a greater capacity for ambidextrous leadership to improve a company's performance. Therefore, indulgence will moderate the relationship between ambidextrous leadership and export performance (Huang and Crotts, 2019). Consequently, organisations in countries with high indulgence have more ability to cope with market dynamics and, therefore, will have an advantage in generating sustainable options for the future (Syed and Malik, 2014). Therefore, we propose the following hypothesis:

*H3* *Countries with a low indulgence factor have a lower moderation effect on the relation between ambidextrous leadership and export performance.*

*H3a* *Countries with a high indulgence factor have a positive moderation effect on the relation between ambidextrous leadership and export performance.*

There are two different perspectives on uncertainty avoidance. One of the most accepted comes from the social sciences: According to Ramirez and Tadesse (2009), aversion to risk is a function of an individual's values and attitudes about a given situation. From the social field, some studies have suggested that the level of uncertainty avoidance is associated with the level of stress that society presents about the image of an unknown future. According to House et al. (2004), these positions support the idea of a level of tolerance for unstructured situations within society.

From the specific field of culture, the attitude towards risk has a social component. Therefore, this component is associated with a country's cultural dynamics that influence individual behaviours and company managers' management styles (Ramirez and Tadesse, 2009). As a result, managers in countries with high levels of uncertainty will be less inclined to take risks. They will consider cash as a factor that allows them to be prepared for unwanted situations in the future. Hofstede (1997) argued that a low level of uncertainty avoidance means a high tolerance for uncertainty. This situation has less ambiguity about the future and is more widely accepted due to the variety of opinions that may arise.

Regarding its effect on ambidextrous leadership and export performance in countries with high uncertainty avoidance, its moderation effect will negatively impact it since it will increase the contingent mechanisms that allow it to manage liquid assets. Such assets are negative debt used to manage crises (Ramirez and Tadesse, 2009). We thus propose the following hypothesis:

*H4* *Countries with a low uncertainty avoidance factor have a lower positive moderation effect in the relation between ambidextrous leadership and export performance.*

*H4a* *Countries with a high uncertainty avoidance factor have a greater positive moderation effect in the relation between ambidextrous leadership and export performance.*

**Figure 1:**
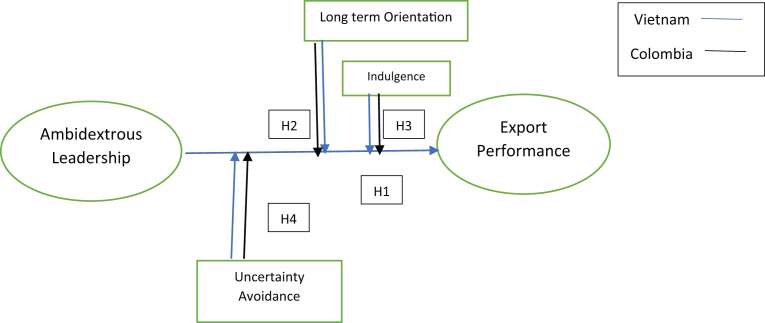
Theoretical model.

Our theoretical model is represented in Figure 1:

## 3 Methodology

In this paper, we collected information from Colombia and Vietnam. Additionally, we developed our questionnaire to apply and identify international businesses, especially BGs in Colombia and Vietnam. We identified 400 BGs in each country, from the following cities: Bogotá, Cali, Medellin, and Barranquilla, to represent BGs in Colombia, and Ho Chi Minh, Hanoi, Da Nang, and Hue for Vietnam.

We developed the questionnaire in English because the original scales were in this language. However, we later changed to Spanish to apply to the BGs in Colombia. For Vietnam, we did not change the language because we detected that the managers were proficient in English. To maintain similar meanings of the items, we contacted grammar professionals and concluded that the scales were not changed in their meanings.

The sample was carried out in person through a survey firm, facilitating that the questionnaires were addressed and answered thoroughly. The approximate time to solve the survey was 15 min, with a response rate of 100% of the selected sample for each country. Participants also signed an informed consent confirming their intention to participate in the study.

The initial descriptive data shows the average Ambidextrous Leadership of BGs across all countries. Our two groups' export performance is relatively similar. Around 80% of BGs in Colombia rely on ambidextrous leadership. Ambidextrous leadership is used by two-thirds of the enterprises in BGs with a high level of export performance.

The BGs in our data were mainly related to the agro-industrial sector (65%), their exports were concentrated in four central destination countries (43%), and they were led predominantly by women (57%). The BGs in Colombia were centred in Bogota and Medellin, representing more than 70% of the exports in Colombia. On the other hand, Ho Chi Minh and Hanoi were Vietnam's most important cities for international operations, with seven main destination countries (54%). They were primarily led by men (63%).

We analysed the invariance of the measurement model following steps developed by Podsakoff et al. (2003): reverse and counterbalance the order of all items, use a similar format for each scale and its items, and check for equal factor loading (χ2 indicator was not significantly different). The questionnaire and its measures did not change.

BG Literature has defined this kind of international firm with three conditions:

1. Firms are founded no more than seven years prior (Jolly et al., 1992; Shrader et al., 2000; Escandon & Hurtado (2012); Escandon et al., 2019).
2. Firms with more than 25% of income come from annual international operations (Escandon et al., 2019; Milanov and Fernhaber, 2009; Taylor and Jack, 2013).
3. Do not have subsidiaries.

### 3.1 Model variables

#### 3.1.1 . Ambidextrous leadership

Employees were asked to rate their bosses' ambidextrous leadership using Zacher and Rosing's (2015) 7-item measure, based on Rosing et al. (2011).'s theory of ambidextrous leadership. "My supervisor checks to make sure that employees follow the right procedures," for example, was one of the things on the list. Other items are related to assessing each person's impression of empowering leadership: “my supervisor makes many decisions with me.”. The exploratory variable showed good reliability (Cronbach's alpha = 0.86).

#### 3.1.2 Export Performance

Jaworski and Kohli (1993) established a compound assessment for export performance that captures the subjective measurement by evaluating general performance relative to key competitors and the objective measurement by evaluating the company's global performance. This variable was rated on a scale of one to seven, with one indicating poor and seven indicating excellent. The results showed a reasonable adjustment for all items in the scale (Cronbach's alpha = 0.91). Export performance has three dimensions: Export Sales and export intensity (It is the perception of profits in terms of monetary worth that is important), Export Growth (exports made over a specific period) and Export profitability (percentage of the firm's total profits in export activities).

#### 3.1.3 Cultural orientation index (The Hofstede model)

Yoo and Donthu (2005) developed the personal cultural orientation scale based on Hofstede's (1980, 2011) dimensions. Their scale is similar to Hofstede's, but it goes beyond work-related cultural values to include cultural values in consumer behaviour. For this paper, we used three dimensions of Hofstede: orientation, restraint vs indulgence, and uncertainty vs avoidance.

### 3.2 Methods

The model has complex relationships between constructs and observed variables relating to the international business field due to running a structural equation system. We used an estimator based on the variance and the calculated models with reflective and formative scales (Henseler, 2017). We used Stata Software to calculate the theoretical model, which allowed us to compare our BGs for each cultural orientation dimension through the Multigroup model (Escandon-Barbosa et al., 2020).

Cultural orientation was the moderating variable between Ambidextrous leadership and Export performance, one item for each dimension. In the case of this research, we calculated the main objective of moderating effects by combining the cultural orientation items with Ambidextrous leadership items.

Due to a structural equation system, the model has complex relationships between the constructs and the observed variables relating to the international business field. It uses an estimator based on variance and calculated models with reflective and formative scales (Henseler, 2017). Stata was used to calculate the theoretical model, which allowed the comparison of born global companies for each dimension of cultural orientation through a multi-group model (Escandon-Barbosa et al., 2020).

The model has a second-order scale (Export Performance), and cultural orientation was the moderating variable with five dimensions developed from Ping's (1996) method. According to this technique, moderating effects must be estimated from the combination of the elements that comprise each latent variable. As a result, Ping (1996) argues that for latent variables X and Z with indicators x1, x2, z1, and z2, a new latent variable XZ must be described with the following combination of the original latent variables: x1z1, x1z2, x2z1, and x2z2 (Escandon-Barbosa et al., 2020). This study considers moderating effects as the main objective, calculated by combining the cultural orientation items with Ambidextrous Leadership items.

### 3.3 Analysis of measurements

We used a confirmatory factorial analysis (CFA) to examine our scales and identify their fits and psychometric measures. It indicated that all measures had acceptable reliability (SCR> 0.8) and a good level associated with Average Variance Extracted (AVE>0.5) (Bagozzi and Yi, 1988; Fornel & Larcker, 1981).

Additionally, the latent variables presented that a significant t-value level generated confirmation of convergent validity (Bagozzi and Yi, 1988). Discriminant validity was confirmed when each scale reported AVE by underlying factors with high levels compared to latent variables with shared variance (Escandon-Barbosa et al., 2019). The resulting statistical fits are as follows: GFI = 0.95; RMSEA = 0.071; SRMR = 0.06; CFI = 0.95; and TLI (NNFI) = 0.95. Table 1 shows the descriptive statistics, SCR, and AVE indicators. The following analysis includes first-order constructions (Ambidextrous leadership and each of the constructs contained in the second order of Export performance: export sales, export growth, and export profitability).

**Table 1:** Descriptive statistics, SCR, and AVE.

		Mean	SD	1	2	3	4	SCR	AVE
1	Ambidextrous leadership	3.61	0.430	1				0.802	0.62
2	Export Sales	4.43	0.33	0.50	1			0.81	0.65
3	Export Growth	4.29	1.031	0.47	0.10	1		0.86	0.70
4	Export profitability	5.08	0.450	0.45	0.05	0.17	1	0.91	0.81

## 4 Results

Our theoretical model showed a good fit and adjustment because all indicator levels satisfied the levels suggested by Anderson and Gerbing (1988): RMSEA = 0.059; CFI = 0.93. Table 2 shows the theoretical model's main results for each proposed hypothesis. For Hypothesis 1, ambidextrous leadership had a positive impact on Export performance in the BGs in Vietnam (β = 0.331; *p* < .01), but the Hypothesis 1a. was rejected in Colombia (β = 0.13; *p* > .1).

**Table 2:** Hypothesis results.

Hypothesis	Coefficient	Colombia Results	Coefficient	Vietnam Results	Differences X2
H1 Ambidextrous Leadership	0.15	Reject	0.331∗∗∗	Accept	Yes
H2 Long Term Moderation	0.173	Reject	0.313∗∗∗	Accept	Yes
H3 Indulgence Moderation	0.281∗∗∗	Accept	0.201∗	Accept	Yes
H4 Uncertainly Avoidance Moderation	0.240 ​∗∗∗	Reject	0.360∗∗∗	Accept	Yes

∗∗∗p < 0.01, ∗∗p < 0.05, ∗ p > 0.1, CFI: 0,94; TLI:0,93; RMSEA: 0.063.

Hypothesis 2 was accepted in Vietnam, which was associated with a long-term orientation, and this orientation had a positive and significant moderation on the relation between Ambidextrous leadership and Export performance (β = 0.313; *p* < .01). However, Hypothesis 2a was rejected in Colombia (β = 0.17; *p* > .1). This shows significant differences in the results of this hypothesis related to Colombia and Vietnam (*t* = 4.60; *p* < .01).

Hypothesis 3, high indulgence— Colombia; and low indulgence — Vietnam—had a positive contribution to Ambidextrous leadership and export performance. However, the moderation was stronger in high indulgence than it was in low indulgence (H3aColombia: β = 0.281; *p* < .01. so, Hypothesis 3 was confirmed; Vietnam: β = 0.201; *p* < .01). Therefore, it was a significant difference between high levels versus low levels of indulgence (*t* = 2.11; *p* < .01) so, Hypothesis 3a was confirmed.

In the country with the higher degree of uncertainty avoidance (Colombia), the relationship between organisational ambidextrous leadership and export performance was significant (β = 0.360; *p* < .01), so Hypothesis 4 was confirmed, but the low level of uncertainty avoidance (Vietnam) was β = 0.24; *p* > .1, so Hypothesis 4a was rejected.

## 5 Conclusions

The main objective of this research was to understand How cultural conditions from Hofstede's perspective influence the relationship between ambidextrous leadership and export performance in BGs in Colombia and Vietnam. In this research, we conducted a moderation effects analysis with three cultural dimensions created by Hofstede: long-term orientation, indulgence, and uncertainty avoidance.

This paper closed different gaps in this field identified in previous research. First, we analysed BGs in two other emerging markets, Latin America and Asia, and we concluded that cultural aspects influence Ambidextrous leadership and their probability of export performance. The second gap analysed the control and use of BG resources to increase their export performance. BGs have different limits related to resources and expertise in international business, and they usually ignore conditions, preferences, and other cultural aspects of their news in international markets. Therefore, their export performance may be affected by their ignorance about cultural differences, such as long-term orientation, indulgence, and uncertainty vs avoidance factors.

The need for the BGS to effectively manage its resources has become more apparent because they have less experience in foreign markets, low-to non-cooperation in their networks, and low, competitive structures. Consequently, BGs need to develop different strategies with their scarce resources, like ambidextrous leadership. When BGs have implemented these strategies, they have managed to increase their Export performance in foreign markets, despite their limited resources, by creating ambidextrous leadership strategies.

According to the literature, ambidextrous leadership is a method that allows a company to carry out stabilisation procedures that allow it to adjust to market situations. As a result, ambidextrous leadership presupposes the presence of managers who aim to harmonise the firm's tendentious contradictions. Similarly, if conflictive behaviours arise, the right-wing leader is to blame.

Another contribution was proving the comparison between Colombia and Vietnam, which have different levels in the three cultural dimensions (time orientation, indulgence vs restraint, and uncertainty vs avoidance). Therefore, in this paper, we used 800 BGs (400 in Colombia and 400 in Vietnam) to participate in our survey in 2019. The model used was a Structural Equation Model to show the moderation effects of Hofstede's cultural dimensions (Ramirez and Tadesse, 2009).

Among the results obtained in the present research, it was possible to verify the existence of the influence of long-term orientation in Asian countries such as Vietnam. This result goes in accordance with Hofstede’s (2021) sustaining this characteristic for this type of culture. On the other hand, countries like Colombia, a Latin American country, tend to have a short-term orientation, giving more priority to factors related to tradition than the search for rewards in the long run.

According to our findings, the relation between ambidextrous leadership and Export performance is strengthened for countries with a long-term orientation trend, such as Vietnam. This relation may be because Eastern countries privilege planning future actions, allowing them to invest in ambidextrous leadership processes (Ocasio, 1997, 2011). Therefore, in countries with high long-term, the leadership will allow the development of capacities more efficient and with more significant planning and order. While for countries with a low long-term orientation (Colombia), the emphasis is on the past and present, generating leadership strategies that cannot count on the time and resources to be carried out by the BG company. In these types of countries, the effect of ambidextrous leadership will be more limited due to its focus on operational activities and sanctions.

In general, long-term orientation generated a substantial difference between the probability of obtaining a good result with ambidextrous leadership strategies and the consequences on BGs’ export performance. BGs in Colombia may have low abilities to implement this strategy, but long-term orientation in Vietnam may support this strategy with more facilities and better results.

The results showed that indulgence had a significant moderating g effect for both countries (Huang & Crotts, 2019). However, this effect was more significant for Colombia than for Vietnam. Countries with a high indulgence like Colombia tend to generate the capacity to face the market dynamics through ambidextrous leadership, which allows them to respond to the environmental necessities. While more restrictive countries, like Vietnam, develop capacities to face the market but should consider planning processes that are long-term oriented more than those that focus on social integration. Therefore, the type of leader the organisation has is essential to focus on the existing strategies in the organisation and achieve greater export results. It is essential to highlight that an ambidextrous leader will be able to understand the market, solve the difficulties of the international environment and guide the collaborators for the organisation's benefit. For countries with high levels of indulgence and low levels, the leader can achieve whether the international results are better or not.

Our results are consistent with Kedmenec and Strašek's (2017), as people from indulgent countries tend to develop operational processes through social activities more than do less indulgent countries. Therefore, the indulgence factor is relevant to obtaining more opportunities to explore ambidextrous leadership because people from indulgence countries are more flexible and able to understand the market dynamics and thus develop leadership with more success (Syed and Malik, 2014).

The moderation effect of uncertainty avoidance was significant in Vietnam but not Colombia. Considering that risk aversion arises because of a country's values, Vietnam is characterised by a low degree of uncertainty avoidance because its emphasis is given to the long-term, considering possible market dynamics in the future and tools in managing resources to face it. In the case of Colombia, uncertainty avoidance is high, which affects the companies' decision-making due to their privileging the use of tools to keep cash, which goes against investments that allow them to have resources and capacities to face market dynamics in the short term.

## 6 Theoretical contribution

From a theoretical perspective, we have tried in this study to understand ambidextrous leadership as a concept that allows us to understand the role of managers in Export performance levels. Additionally, the results showed the possibility of identifying the implications for BGs' Export performance (Khanna and Palepu, 1997). Additionally, we looked at how BGs use the balance of their leadership style considering cultural contexts such as Hofstede's dimensions. We carried out the leadership of cultural dimensions that have been little studied in the field of BG, which has allowed us to identify dynamics of great importance to ensuring BGs' success in new markets.

Our results also confirm the results of authors such as Jia et al. (2021) on the interaction between ambidextrous leadership and its impact on BGs’ ability to meet market demands related to business performance. In the same way, it responds to a latent need to investigate the different dimensions and influences of ambidextrous leadership, especially resilience.

## 7 Country policy contribution and limitations

Within the policy, recommendations are the need for governmental support mechanisms for business development through programs that improve competitiveness through human capital development, particularly in positions of leadership or focusing on internalisation of the company. The business development programs aim to strengthen a company's role in the international market and promote international collaboration. Furthermore, the need for government interventions to improve private investment through developing industrial development mechanisms for the future is identified as one of the main disadvantages in countries such as Colombia and Vietnam.

On the other hand, businesses must strengthen long-term strategies that include human, technical, and financial resources. Managers must consider their vision and decisions due to their involvement in strategy and the organisation's national and international success.

Finally, our research has relevant contributions, but at the same time, it has limitations that we recognise. First, our data is for BGs in Colombia and Vietnam, collected over one year (transversal model). Future research lines, then, will be needed to confirm this model with data series to determine if cultural dimensions are relevant to the relationship between ambidextrous leadership and Export performance or if they will lose explanatory power. Another future line is conducting comparative research between BGs and another international venture to analyse differences among cultural dimensions and firms.

## Declarations

### Author contribution statement

Diana Escandon: Conceived and designed the experiments; Performed the experiments; Analyzed and interpreted the data; Wrote the paper.

Jairo salas: Conceived and designed the experiments; Performed the experiments; Contributed reagents, materials, analysis tools or data; Wrote the paper.

### Funding statement

JairoSalas-Paramo was supported by Toward and evolutionary project in Pontificia Universidad Javeriana- Cali. 020100781.

### Data availability statement

The authors do not have permission to share data.

### Declaration of interest’s statement

The authors declare no conflict of interest.

### Additional information

No additional information is available for this paper.
